# Rare-Earth Activated Nitride Phosphors: Synthesis, Luminescence and Applications

**DOI:** 10.3390/ma3063777

**Published:** 2010-06-21

**Authors:** Rong-Jun Xie, Naoto Hirosaki, Yuanqiang Li, Takashi Takeda

**Affiliations:** Nitride Particle Group, Nano Ceramics Center, National Institute for Materials Science, Namiki 1–1, Tsukuba, Ibaraki 305–0044, Japan; E-Mails: Hirosaki.Naoto@nims.go.jp (N.H.); (Y.Q.L.); Takeda.Takashi@nims.go.jp (T.T.)

**Keywords:** nitride, luminescence, LED

## Abstract

Nitridosilicates are structurally built up on three-dimensional SiN4 tetrahedral networks, forming a very interesting class of materials with high thermomechanical properties, hardness, and wide band gap. Traditionally, nitridosilicates are often used as structural materials such as abrasive particles, cutting tools, turbine blade, *etc*. Recently, the luminescence of rare earth doped nitridosilicates has been extensively studied, and a novel family of luminescent materials has been developed. This paper reviews the synthesis, luminescence and applications of nitridosilicate phosphors, with emphasis on rare earth nitrides in the system of M-Si-Al-O-N (M = Li, Ca, Sr, Ba, La) and their applications in white LEDs. These phosphors exhibit interesting luminescent properties, such as red-shifted excitation and emission, small Stokes shift, small thermal quenching, and high conversion efficiency, enabling them to use as down-conversion luminescent materials in white LEDs with tunable color temperature and high color rendering index.

## 1. Introduction

Covalent nitridosilicate materials, such as Si_3_N_4_ and its solid solutions, have been long known as structural ceramics due to their excellent thermo-mechanical properties, high strength, high hardness, high chemical stability, *etc*. The promising physical properties of nitridosilicate ceramics are predominantly dependent on the stiff crystal structure that is generally built up on three-dimensional SiN_4_ tetrahedral networks [1,2]. The degree of condensation of the network SiX4 tetrahedra can be simply evaluated by the ratio of tetrahedral Si centers and bridging atom X. In oxosilicates, the Si:X ratio reaches the maximum of 0.5 in SiO_2_, while in nitridosilicates the Si:X ratio may vary in a broad range of 0.25–0.75, implying highly condensed SiX4 tetrahedral networks can be achieved in nitridosilicates. Consequently, the high degree of condensation and the high stability of chemical bonding between the constituent elements lead to extraordinary chemical and thermal stability of nitridosilicates. 

Extensive investigations have been carried out on the mechanical properties of covalent nitride materials and their applications as structural components, abrasive particles, and anti-corrosion parts. Recently, very interesting luminescence has been observed in rare-earth-doped nitridosilicate materials, which opens up totally new application fields for traditional nitridosilicate materials as phosphors in lamps, display, *etc*. [3,4,5,6,7,8,9,10,11,12]. Definitely, a novel family of luminescence materials—rare earth activated nitridosilicates—has been formed, and is greatly beneficial to the development of advanced lighting systems such as white light-emitting diodes (LEDs). In 1998, Krevel *et al*. [5] observed long-wavelength emissions in Ce^3+^-doped Y-Si-O-N compounds, and the luminescence of Ce^3+^ was strongly related to the O/N ratio. Hoppe *et al.* [4] found that, the excitation and emission of Eu^2+^ was significantly red-shifted in Ba_2_Si_5_N_8_, and an orange emission color was observed. A large red-shift in emission and excitation bands was also seen in Eu^2+^-doped α-sialon, resulting in a yellow emission color under the blue light irradiation [6,7,9,11]. The red-shifted luminescence of rare earth ions with 5d–4f electronic transitions in nitride materials is ascribed to large crystal-field splitting and the strong nephelauxetic effect. The large crystal field splitting is due to the large formal charge of N^3-^ compared to O^2-^; and the nephelauxetic effect is attributed to the strong covalent chemical bonding between rare earth ions and ligand. 

There were no suitable applications except pigment for nitride phosphors until the solid state lighting, *i.e.*, white LEDs, came on stage and subsequently developed very fast in recent years. White LEDs, combining LED chips with wavelength-conversion materials to create white light, promise high brightness, high efficiency, low electrical power consumption, small volume, long lifetime, high response speed, and low maintenance, and are thus considered as the next-generation solid state lighting replacing traditional incandescent and fluorescent lamps. Generally, there are three ways to generate white light in phosphor-converted LEDs (pc-LEDs): (i) ultraviolet LED chip + red, green and blue (RGB) phosphors; (ii) blue LED chip + yellow phosphor; and (iii) blue LED chip + RG phosphors. As current ultraviolet (UV) LEDs have relatively low quantum efficiency and brightness, and the degradation or damage of packaging materials and illuminated bodies occurs by UV light irradiation, using blue LEDs as the primary lighting source is therefore the mainstream of pc-LEDs. Anyhow, no matter what kind of LED chips are used, phosphors for white LEDs should have high conversion efficiency of UV or blue light, and emit suitable colors with high brightness. 

This article reviews the synthesis, photoluminescence, and applications of nitride-based phosphors that have been reported recently. 

## 2. Synthesis of Nitride Phosphors

The starting materials for the preparation of nitride phosphors usually consist of nitrogen-containing materials such as Si_3_N_4_, AlN, M_x_N_y_ (M = alkaline earth metals), which requires the nitrogen atmosphere to protect the oxidation of the powder and to suppress the decomposition of powders at high temperatures. In addition, nitride raw materials have low reactivity due to their low diffusion coefficient, which indicates the necessity of high firing temperatures (usually >1300 °C). These requirements enable the solid state reaction method to be the dominant synthetic route to nitride phosphors. On the other hand, considering the high cost of raw nitride powders and the high firing temperatures, some low-temperature synthesis approaches have been employed by using oxide or ammonometallate starting materials [13,14,15,16]. These approaches include gas reduction and nitridation (GRN) [13], carbothermal reduction and nitridation (CRN) [14], and high pressure ammothermal methods [15,16]. Furthermore, these methods are able to produce fine or nano-sized phosphor particles that are difficult to obtain by the solid state reaction. In this Section, the synthetic methods for nitride phosphors will be reviewed. 

### 2.1. Solid State Reaction

This method involves chemical reactions among solid constituents of raw powders at high temperatures, which includes following steps: (i) diffusion at interfaces of solid particles; (ii) chemical reaction at atomic levels; (iii) nucleation; and (iv) material transportation and growth of nuclei. Whether a step is diffusion- or reaction-controlled, the starting materials play a key role in determining processing parameters such as heating rate, temperature, holding time, atmosphere, *etc*. 

For alkaline metal nitridosilicates, there are several options of silicon-source or alkaline source. For silicon source, Si_3_N_4_ is usually used as it is stable in ambient air and commercially available. For example, α-sialon is prepared by the following reaction at approximately 1700 °C [6,7,9,11].

(12-m-n)/3Si_3_N_4_ + (2n-m)/6Al_2_O_3_ + (4m+n)/3AlN + m/2CaO

→ Ca_m/2_Si_12-m-n_Al_m+n_O_n_N_16-n_ (1)

However, the reactivity of Si_3_N_4_ is so low that high temperature is usually applied. In addition, the surface oxide layer in Si_3_N_4_, *i.e*., SiO_2_, inevitably introduces oxygen contamination into the pure nitride phosphor products. Alternatively, silicon diimide, Si(NH)_2_, is also used as a silicon source [4,17,18], which decomposes into Si_3_N_4_ by releasing hydrogen and nitrogen gases: 3Si(NH)_2_ → Si_3_N_4_ + 3H_2_ +N_2_. Si(NH)_2_ has higher reactivity than Si_3_N_4_, and thus the firing temperature can be reduced. For example, Sr_2_Si_5_N_8_ was synthesized by reacting strontium metal with silicon diimide in Schnick’s group as below [18]:

2Sr + 5Si(NH)_2_ → Sr_2_Si_5_N_8_ + 5H_2_ + N_2_ (2)

For alkaline-earth sources, alkaline-earth carbonates, metals or nitrides are usually adopted, the latter two are extremely air-and moisture-sensitive. Alkaline-earth nitrides can be synthesized by nitridation of corresponding metals in N_2_ or H_2_-N_2_ at 800–1000 °C. Sr_2_Si_5_N_8_ was also prepared in Hintzen’s group by using the following reaction [19]:

2Sr_3_N_2_ + 5Si_3_N_4_ → 3Sr_2_Si_5_N_8_ (3)

For the preparation of nitride phosphors by the solid state reaction, three types of synthetic facilities including gas-pressure sintering furnace, radio-frequency furnace, and horizontal tube furnace are employed predominantly. The gas-pressure sintering furnace enables the application of nitrogen gas pressure in the range of 0.1–1.0 MPa, whereas the other two systems promise the ambient nitrogen atmosphere.

Using the solid state reaction method can produce phosphor particles with high crystallinity, large size and high luminescence. Moreover, this method is suitable for mass production.

### 2.2. Gas Reduction and Nitridation (GRN)

As the phosphor prepared by the solid state reaction is usually agglomerated, the particle size, as well as the particle size distribution, needs to be controlled by the post-treatment such as pulverization. The phosphor particles might be damaged by the pulverization process, which would form surface defects or traps that quench the luminescence. In addition, some raw materials, such as alkaline-earth metals or nitrides, are air sensitive and expensive, yielding a complex and multi-step process for the preparation of nitride phosphors. Therefore, it is necessary to find some simple and cost-effective ways to synthesize phosphors with uniform and fine particle size, and high luminescence. 

The gas reduction and nitridation method is one effective way to prepare binary nitrides such as AlN or GaN, through the chemical reaction between NH_3_ and Al_2_O_3_ or Ga_2_O_3_. Recently, multi-nary nitride phosphors have been synthesized by the GRN method. Suehiro *et al*. [13] reported that fine yellow-emitting Ca-α-sialon: Eu^2+^ powders (~300 nm) could be obtained by firing the precursor materials in the system of CaO-SiO_2_-Al_2_O_3_-Eu_2_O_3_ at 1400–1550 °C in a flowing NH_3_-CH_4_ gas mixture. In this case, the gas mixture of NH_3_ and CH_4_ acts as both a reducing and nitriding agent. Ce^3+^-doped LaSi_3_N_5_ blue-emitting phosphors were also synthesized by using the oxide precursors of La_2_O_3_-SiO_2_-CeO_2_ [20]. In addition, nitride phosphors such ash CaSiN_2_ or Sr_2_Si_5_N_8_ were prepared by nitriding alkaline-earth silicides in a flowing NH_3_ gas [21,22].

The phosphors prepared by the GRN method are usually loose, and exhibit fine particle sizes, and a narrow size distribution. Furthermore, the firing temperature can be lowered by about 100–200 °C compared to that used in the solid state reaction method. 

### 2.3. Carbothermal Reduction and Nitridation (CRN)

Carbothermal reduction is a well known process to reduce oxides by heating them in the presence of carbon. Carbothermal reduction nitridation (CRN) is used to produce nitrides or oxonitrides from an oxide using the overall reaction:

3M_x_O + 3C + N_2_ → M_3x_N_2_ + 3CO (4)

For example, Si_3_N_4_ powders were synthesized by heating the powder mixture of carbon and silica in a nitrogen atmosphere. 

Recently, the CRN method was attempted to prepare multi-nary nitride or oxynitride phosphors. Red-emitting M_2_Si_5_N_8_:Eu^2+^ (M = Ca, Sr) phosphors were obtained by firing the mixture of MCO_3_ (M = Sr, Ca), Si_3_N_4_, Eu_2_O_3_, and fine graphite at 1550 °C in flowing nitrogen atmosphere [14]. The lowering of oxygen content of the phosphor is possible with this method.

The phosphors synthesized by the CRN method have good crystallinity and a narrow particle size distribution. The carbon content should be carefully controlled, as residual carbon will reduce the luminescence of the products.

### 2.4. Ammothermal Method

The ammothermal method is a solution process that can synthesize nitride compounds at relatively low temperatures. It is analogous to the route for the synthesis of oxide materials in an aqueous solution, which may produce nano-sized materials with a variety of morphologies and may avoid the need for high temperatures and high pressures.

Li *et al.* [15] reported the synthesis of CaAlSiN_3_: Eu^2+^ by a metal-ammonometallate-nitride route. Nanocrystalline Eu^2+^-doped CaAlSiN_3_ was prepared by heating the mixture of Ca-Eu-Al-Si alloy and sodium amide up to 800 °C in 100 MPa supercritical ammonia. The synthesis temperature in this method was about 1000 °C lower than that used in solid state reaction. Furthermore, a variety of forms, such as powders, films, and single crystals, might be obtained by controlling the processing parameters and precursor materials. 

Zeuner *et al.* [16] applied a similar method to produce red-emitting M_2_Si_5_N_8_:Eu^2+^ (M = Ca, Sr, Ba) at low temperatures. The synthesis procedure included the preparation of metal amide precursors (M(NH_2_)_2_, M = Sr, Ba, Eu) by dissolving metals in supercritical ammonia, and then the reaction between metal amides and silicon diimide in a radio-frequency furnace at 1150–1400 °C. The shape and size of M_2_Si_5_N_8_:Eu^2+^ phosphors were altered by controlling the temperatures, annealing times, cooling rates, and precursors, and spherical powders could be achieved by prolonging annealing times.

## 3. Photoluminescence Properties of Nitride Phosphors

The structural versatility in nitride materials promises the abundant emission colors of nitride phosphors when rare earth ions such as Eu^2+^ or Ce^3+^, exhibiting the electronic transitions of 4f to 5d, are introduced as luminescence centers. In addition, either the strong covalent bonding between rare earth and ligand (nephelauxetic effect) or the large crystal-field splitting results in the significant red-shift of excitation and emission spectra, enabling nitride phosphors to absorb near UV or blue light strongly. Moreover, small Stokes shifts usually observed in nitride phosphors lead to high conversion efficiency as well as small thermal quenching. These interesting features of nitride phosphors make them very suitable for use as down-conversion materials in white LEDs. 

In this Section, the luminescent properties of Eu^2+^- or Ce^3+^-doped nitride phosphors, such as excitation and emission spectra, quantum efficiency, and thermal quenching, will be reviewed.

### 3.1. Blue-emitting Phosphors

#### 3.1.1. Ca-α-sialon:Ce^3+^

**Figure 1:**
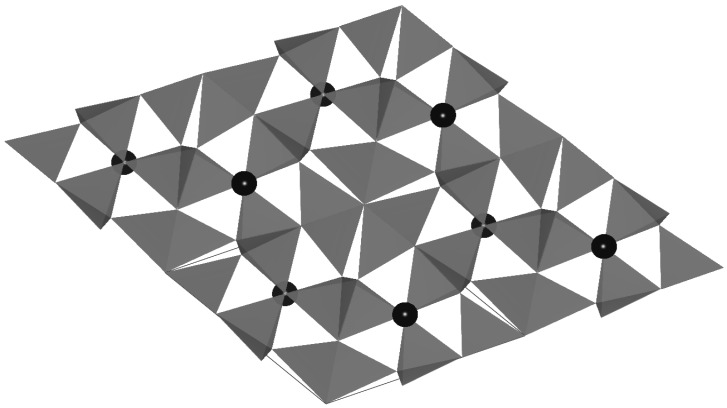
The crystal structure of Ca-α-sialon viewed from [001].The black balls represent the Ca atoms.

α-sialon is a solid solution of α-Si_3_N_4_, which is formed by the partial replacement of Si-N bonds with Al-N and Al-O bonds. A general formulae of α-sialon, is given as M_x_Si_12-m-n_Al_m+n_O_n_N_16-n_ (x is the solubility of the M metal, *m* and *n* are the numbers of Al-N and Al-O bonds substituting for Si-N bonds, respectively) [23]. The charge discrepancy caused by the substitution is compensated by the introduction of M cations, such as Li^+^, Mg^2+^, Ca^2+^, Y^3+^ and some lanthanides. The M cations occupy the interstitial sites in the α-sialon structure, which are coordinated by seven (N, O) anions (Figure 1). 

**Figure 2:**
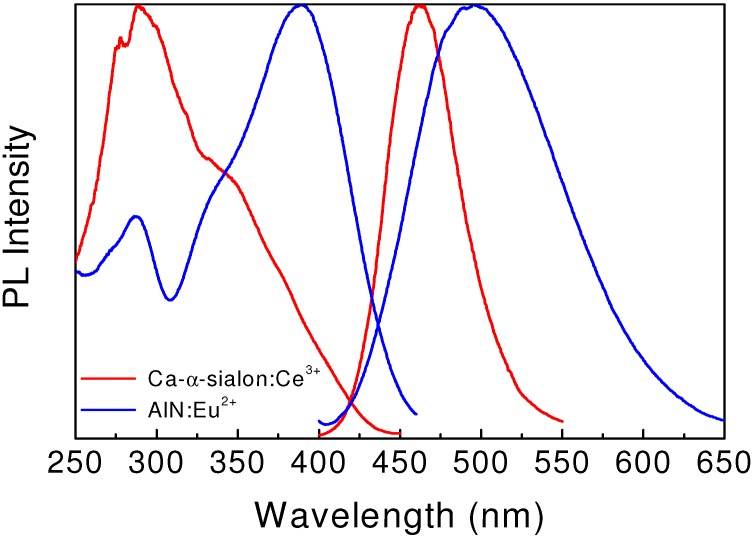
Excitation and emission spectra of blue-emitting Ca-α-sialon:Ce^3+^ and AlN:Eu^2+^.

By doping α-sialon with some rare earth ions including Eu^2+^, Ce^3+^, Tb^3+^, Pr^3+^, Sm^3+^, Yb^2+^, and Dy^3+^, a variety of emission colors can be observed due to the 5d → 4f or 4f → 4f electronic transitions of rare earth ions [6,7,9,10,11,24]. Among these, the Ce^3+^-doped α-sialon shows an intense blue emission upon the UV- or near-UV excitation [6,10]. The emission spectrum, centered at 495 nm, extends from 400–650 nm (Figure 2). Moreover, the emission of α-sialon: Ce^3+^ can also be tuned in the range of 485–505 nm by tailoring the chemical composition or by varying the Ce^3+^ concentration. The excitation spectrum shows a broad band with the peak located at 390 nm, which matches well with the emission wavelengths of UV or near-UV LEDs.

#### 3.1.2. LaAl(Si_6-z_Al_z_)N_10-z_O_z_:Ce^3+^

LaAl(Si_6-z_Al_z_)N_10-z_O_z_ (JEM) has an orthorhombic structure (space group *Pbcn*) [25]. The Al atoms and the (Si,Al) atoms are tetrahedrally coordinated by (N,O) atoms, yielding an Al(Si,Al)_6_(N,O)_10_^3-^ network. The La atoms are accommodated in the tunnels extending along the [001] direction and are irregularly coordinated by seven (N,O) atoms at an average distance of 2.70 Å. The emission spectrum of Ce^3+^-doped JEM displays a broad band covering from 400 to 700 nm under the 368 nm excitation, with the peak located at 475 nm [26]. The broad excitation spectrum is due to the allowed 4f → 5d electronic transition of Ce^3+^, extending from 200 to 450 nm. Both the excitation and emission spectra are red-shifted when the concentration of Ce^3+^ or the *z* value increases, enabling this blue phosphor to be excited efficiently by UV (370–400 nm) or near-UV (400–410 nm) LEDs. The external quantum efficiency of JEM: Ce^3+^ is about 45% when measured under the 405 nm excitation.

#### 3.1.3. AlN:Eu

Hirosaki *et al.* [27] reported the cathodoluminescence of Eu^2+^-doped AlN. A strong blue emission was observed in AlN:Eu^2+^ upon the electron beam irradiation, which enables AlN:Eu^2+^ to be used as a blue phosphor for field-emission displays. Inoue *et al.* [28] studied the photoluminescent properties of AlN:Eu^2+^, and found that it was also an interesting blue phosphor for white LEDs. As shown in Figure 2, AlN:Eu^2+^ shows a broad emission band centered at 465 nm upon irradiation of the UV light (280–380 nm). It has low thermal quenching, retaining the luminance of 90% at 150 °C. The absorption and external quantum efficiency of the product is 63% and 46% at an excitation wavelength of 365 nm, respectively. The excellent luminescent properties and thermal stability enable AlN:Eu^2+^ to be an potential blue-emitting down conversion phosphor in white LEDs utilizing InGaN UV-LED chips.

### 3.2. Green-emitting Phosphors

#### 3.2.1. β-sialon:Eu^2+^

β-sialon, with the chemical composition of Si_6-z_Al_z_O_z_N_8-z_ (0<z<4.2), is solid solutions of β-Si_3_N_4_ [29]. It forms by the partial replacement of Si-N with Al-O bonds, and z means the substitution number of Al-O or the solubility of Al in the lattice. 

**Figure 3:**
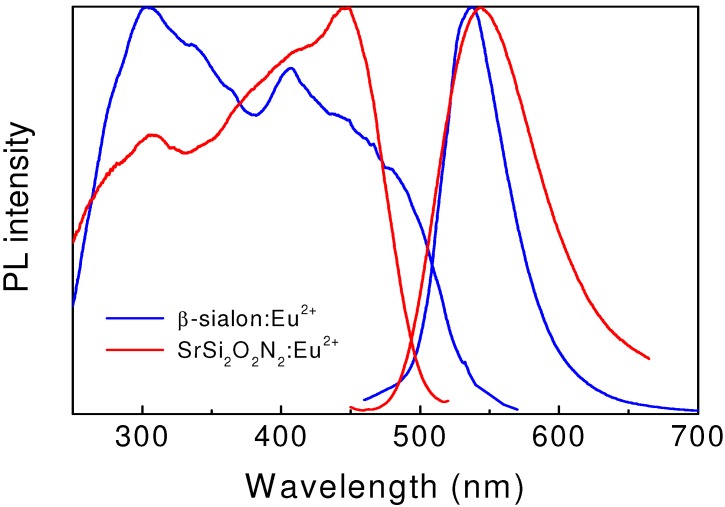
Excitation and emission spectra of green-emitting β-sialon:Eu^2+^ and SrSi_2_O_2_N_2_:Eu^2+^.

By doping β-sialon with a small amount of Eu^2+^, Hirosaki *et al.* [30] observed an intense green emission color upon UV or visible light excitation, which was ascribed to the allowed 5d → 4f transitions of Eu^2+^. As seen in Figure 3, the emission spectrum consists of a single broadband with a maximum at 535 nm and a full width at half maximum of 55 nm. Two well-resolved broadbands, centered at 303 and 400 nm, and a number of shoulders are observed in the excitation spectrum. The internal quantum efficiency of the β-sialon:Eu^2+^ phosphor is 70, 54 and 50% at the excitation wavelength of 303, 405 and 450 nm, respectively; and the corresponding external quantum efficiency is 61, 41and 33%. In addition, β-sialon:Eu^2+^ has low thermal quenching, with its luminescence retaining 90% at 150 °C [31].

The location of Eu^2+^ in β-sialon is under debate, because it is generally accepted that β-sialon cannot accommodate rare earth ions. Recently, Kimoto *et al.* [32] directly observed the Eu^2+^ ions in β-sialon by STEM. He addressed that Eu^2+^ ions were located in the channels along the c-direction, which confirms that the green emission is originated from Eu^2+^ in β-sialon rather than defects or impurity phases. 

#### 3.2.2. SrSi_2_O_2_N_2_:Eu^2+^

The crystal structure of SrSi_2_O_2_N_2_ was investigated by Schnick’s group [33]. It crystallizes in the triclinic structure with the space group of *P*1. The lattice parameters are shown as below: *a* *=* 7.0802 Å, *b* *=* 7.2306 Å, *c* *=* 7.2554 Å, *α* *=* 88.767°, *β =* 84.733°, *γ =* 75.905° and *V =* 358.73 Å^3^, *Z =* 4. SrSi_2_O_2_N_2_ are structurally related to CaSi_2_O_2_N_2_, both representing a class of layered materials with layers of (Si_2_O_2_N_2_)^2^^-^ that consist of SiON^3-^ tetrahedrons [34]. The N atom bridges three Si atoms, while the O atom is bound terminally to the Si atom. There are four types of sites for the Sr^2+^ ions, each surrounded by six oxygen atoms in a distorted trigonal prismatic manner.

Eu^2+^-doped SrSi_2_O_2_N_2_ shows a broad emission band peaked at 540 nm with FWHM of 82 nm [35,36]. The excitation spectrum of SrSi_2_O_2_N_2_:Eu^2+^ shows two well-resolved broadbands centered at 300 and 450 nm (Figure 3). This green phosphor has a conversion efficiency of about 90%, and also exhibits a low thermal quenching, which allows it to be used in white LEDs with blue LEDs as a primary lighting source. Moreover, the emission band of SrSi_2_O_2_N_2_:Eu^2+^ can be tuned by substitution of Sr^2+^ by either Ca^2+^ or Ba^2+^. Partial replacement of Sr^2+^ by Ca^2+^ or Ba^2+^ leads to a red-shifted Eu^2+^ emission [37]. For CaSi_2_O_2_N_2_:Eu^2+^ and BaSi_2_O_2_N_2_:Eu^2+^, the Eu^2+^ emission is about 560 and 500 nm, respectively [35].

#### 3.2.3. Sr_3_Si_13_Al_3_O_2_N_21_:Eu^2+^

The structure of Sr_3_Si_13_Al_3_O_2_N_21_ was reported by Fukuda *et al*. [38]. It has an orthorhombic structure with the lattice parameters of a = 9.037, b = 14.734, and c = 7.464 Å. The Sr^2+^ ion is coordinated by 10 (N,O) anions, with the average distance between two Sr^2+^ ions of ~3 Å.

Eu^2+^-doped Sr_3_Si_13_Al_3_O_2_N_21_ emits an intense green emission upon the UV or blue light excitation, and shows a single broad band with FWHM ~66 nm. The emission peak varies in the range of 515–525 nm. The excitation spectrum also exhibits a broad band, extending from UV region to 500 nm. Under the 460 nm excitation, Sr_3_Si_13_Al_3_O_2_N_21_:Eu^2+^ has an external quantum efficiency of 67%. In addition, the thermal quenching of this green phosphor is small, due to the rigid network of (Si,Al)(O,N)4 tetrahedra in its crystal structure.

#### 3.2.4. Ba_3_Si_6_O_12_N_2_:Eu^2+^

The structure of Ba_3_Si_6_O_12_N_2_ was reported by the Mitsubishi Chemical Group [39]. It has a trigonal structure with the space group of P3, the lattice parameters of which are a = 7.5046 and c = 6.4703 Å. The compound is built up of corner sharing SiO_3_N tetrahedra forming corrugated layers between which Ba^2+^ ions are located. The Ba^2+^ ions occupy two different crystallographic sites; one is trigonal anti-prism with six oxygen atoms, and the other is trigonal anti-prism with six oxygen atoms and one nitrogen atom, which is further capped with one nitrogen atom.

Eu^2+^-doped Ba_3_Si_6_O_12_N_2_ shows a green color with a broad emission band centered at 525–530 nm. The full width at half maximum of the emission band is about 68 nm. The excitation spectrum displays a broad band covering from 250 to 500 nm. In addition, the emission intensity of Ba_3_Si_6_O_12_N_2_:Eu^2+^ at 100 °C is about 90% of that measured at room temperature, whereas it is about 75% for (Ba, Sr)_2_SiO_4_:Eu^2+^.

### 3.3. Yellow-emitting Phopshors

#### 3.3.1. α-sialon:Eu^2+^

Eu^2+^-doped Ca-α-sialon shows intense yellow emission color when it is excited by blue light [6,7,9,10,12]. It thus offers an alternative way to create white light by using Ca-α-sialon:Eu^2+^ and a blue LED chip. The excitation spectrum of Ca-α-sialon:Eu^2+^ consists of two broadbands centered at 300 and 420 nm, respectively. The broadband emission spectrum, with the peak wavelength of 580 nm, covers from 500 to 750 nm with the full width of half maximum of 94 nm. The external quantum efficiency is about 60% upon the 450 nm excitation. The temperature-dependent luminescence shows that Ca-α-sialon:Eu^2+^ has low thermal quenching, the luminescence of which can be maintained at 90% of that measured at 150 °C [40].

**Figure 4:**
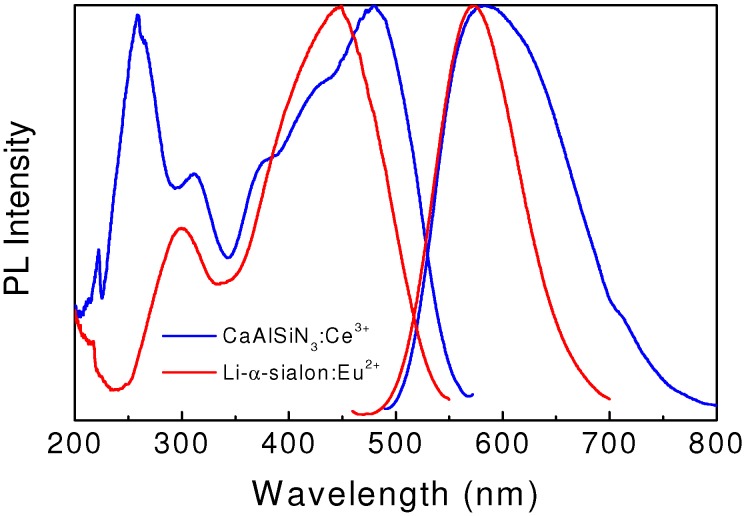
Excitation and emission spectra of yellow-emitting Li-α-sialon:Eu^2+^ and CaAlSiN_3_:Ce^3+^.

The emission color of α-sialon:Eu^2+^ can be tuned in a wide range of 565–605 nm, by tailoring the composition of the host lattice and controlling the concentration of Eu^2+^ [40,41,42]. The emission band is blue-shifted by replacing Ca with Li [40,41], and red-shifted by substituting Ca with Y [42]. Typically, a green-yellow emission color can be achieved in Li-α-sialon:Eu^2+^ (Figure 4), which promises the fabrication of white light with high color temperatures [41].

#### 3.3.2. CaAlSiN_3_:Ce^3+^

CaAlSiN_3_ has the orthorhombic structure with the *Cmc*21 space group, in which the Ca atom has one crystallographic site at 4a, located at the channels built up by the six-membered rings of the (Si, Al)N4 tetrahedra and directly coordinated by nitrogen atoms with a lower coordination number (CN = 5) crossed over the unit cell in the range of 2.8 Å [43,44]. The Al and Si atoms are randomly distributed on the same tetrahedral sites and connected with N atoms to form the vertex-linked M6N18 rings (M = Al, Si).

As shown in Figure 4, CaAlSiN_3_:Ce^3^^+^ emits efficient yellow-orange light with a maximum at around 580 nm upon the UV and/or blue light excitation [44]. The FWHM value of the broad emission band is around 140 nm, which is characteristic of the 5d → 4f electronic transition of Ce^3+^. The external quantum efficiency is 54% under the 450 nm excitation. The emission intensity at 150 °C is about 87% of that measured at room temperature. 

The yellow-orange emission color and the broad emission band of CaAlSiN3:Ce^3^^+^ enable it to produce warm white with high color rendering index. 

### 3.4. Red-emitting Phosphors

#### 3.4.1. M_2_Si_5_N_8_:Eu^2+^ (M *=* Ca, Sr, Ba)

The crystal structure of M_2_Si_5_N_8_ (M *=* Ca, Sr, Ba) was investigated by Schnick *et al*. [17,18] Ca_2_Si_5_N_8_ has a monoclinic crystal system with the space group of *Cc*, and both Sr_2_Si_5_N_8_ and Ba_2_Si_5_N_8_ have an orthorhombic lattice with the space group of *Pmn2_1_*. The local structure is quite similar for these crystals: half of the nitrogen atoms connect two Si neighbors and the other half of the nitrogen atoms have three Si neighbors. Each Ca atom in Ca_2_Si_5_N_8_ is coordinated to seven nitrogen atoms, while Sr in Sr_2_Si_5_N_8_ and Ba in Ba_2_Si_5_N_8_ are coordinated to eight or nine nitrogens.

**Figure 5:**
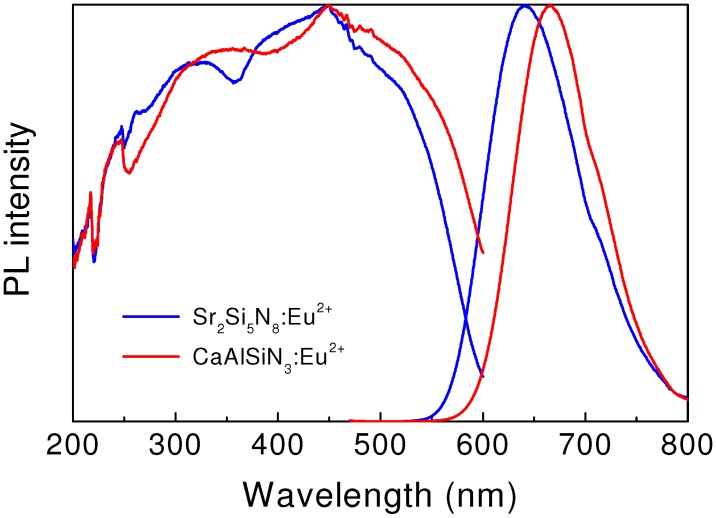
Excitation and emission spectra of red-emitting Sr_2_Si_5_N_8_:Eu^2+^ and CaAlSiN_3_:Eu^2+^.

The luminescence of Eu^2+^ in M_2_Si_5_N_8_ (M = Ca, Sr, Ba) was reported by Hoppe (M = Ba) and Li (M = Ca, Sr, Ba), respectively [4,19]. The emission color of these materials varies from orange to red, depending on the type of alkaline earth metals. The peak emission wavelength is 623, 640 and 650 nm for Ca_2_Si_5_N_8_:Eu^2+^, Sr_2_Si_5_N_8_:Eu^2+^, and Ba_2_Si_5_N_8_:Eu^2+^, respectively. Figure 5 shows the excitation and emission spectra of Sr_2_Si_5_N_8_:Eu^2+^. It indicates that the emission band is red-shifted with increasing ionic size of alkaline earth metals. The broad excitation spectrum centered at 450 nm extensively shifts to the long wavelength side and covers the visible light range. 

The external quantum efficiency of Sr_2_Si_5_N_8_:Eu^2+^ is ~64% when it is excited at 450 nm [45]. The emission intensity of this red phosphor remains 86% of that measured at room temperature, indicative of low thermal quenching.

#### 3.4.2. CaAlSiN_3_:Eu^2+^

The luminescence of Eu^2+^ in CaAlSiN_3_ was reported by Uheda *et al*. [43] CaAlSiN_3_:Eu^2+^ is a red-emitting phosphor. As see in Figure 5, the excitation spectrum is extremely broad and covers the spectral range of 250–600 nm, matching well with the emission wavelength of near UV or blue LEDs. A broad emission band centered at 650 nm is observed upon the 450 nm excitation. The emission intensity of CaAlSiN_3_:Eu^2+^, measured at 150 °C, is about 89% of that measured at room temperature.

The emission color of CaAlSiN_3_:Eu^2+^ can be blue-shifted by the partial substitution Sr for Ca, which improves the luminous efficiency of white LEDs [46].

## 4. White LEDs Using Nitride Phosphors

Phosphors play a key role in determining the color temperature, chromaticity coordinates, color rendering index, luminous efficiency, and lifetime of phosphor-converted white LEDs. The first white LED was fabricated by combining a yellow-emitting garnet phosphor (YAG:Ce^3+^) and a blue LED chip. Although this dichromatic white LED has been widely used as mobile phone backlights, indicators, and flash lighting, its low color rendering index (Ra < 80) is not acceptable for general lighting. In addition, warm white cannot be created by only using the YAG:Ce^3+^ phosphor due to the lack of red component in the spectral region. As nitride phosphors excited by blue light have a variety of emission colors depending on the activator ions or the composition of host crystals, warm white LEDs using single phosphors or even high color rendering white LEDs can be achieved by combining them with blue LED chips. In addition, as nitride phosphors are generally thermally stable or have low thermal quenching, small variations in the color point or small degradation in the performance of white LEDs will be expected. In this Section, the applications of nitride phosphors in white LEDs are reviewed.

### 4.1. One-phosphor-converted (1-pc) LEDs

The first white LED using nitride phosphors was reported by Sakuma *et al*. [11]. By using an orange-yellow Ca-α-sialon:Eu^2+^ with the peak emission wavelength of 585 nm and a blue LED chip (λ_em_ = 450 nm), a warm white LED was obtained. The white LED has the correlated color temperature of ~2750 K, and the color rendering index of ~60. Moreover, white LEDs using Ca-α-sialon:Eu^2+^ have smaller changes in chromaticity coordinates than those using YAG: Ce^3+^. 

**Figure 6:**
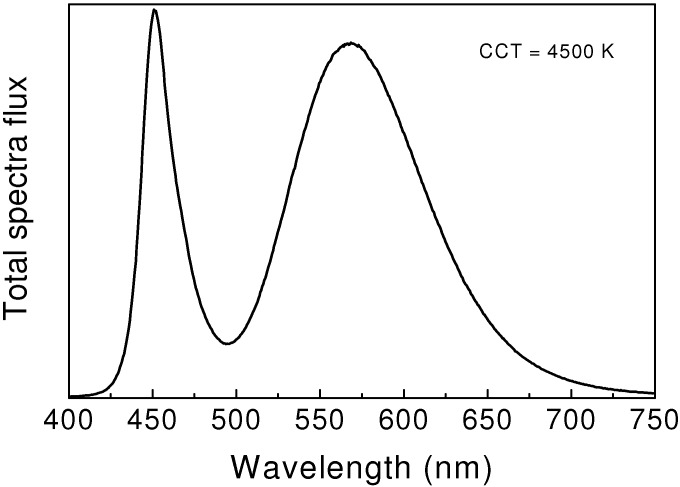
Emission spectrum of 1-pc white LEDs using a Li-α-sialon:Eu^2+^ and a blue LED chip.

As Ca-α-sialon:Eu^2+^ has the orange-yellow emission color, this makes it impossible to create white light with high color temperature. To achieve this goal, Li-α-sialon:Eu^2+^ with short emission wavelengths was developed by our group, which emits green-yellow colors [41]. Thus, white LEDs, having the color temperature of 5200 K, can be made by using Li-α-sialon:Eu^2+^ (Figure 6). The luminous efficacy of white LEDs is 46–55 lm/W, which is two or three times higher than that of incandescent lamps. 

Warm white LEDs can also be fabricated by using the orange-yellow CaAlSiN_3_:Ce^3+^ and a blue LED chip (λ_em_ *=* 450 nm). Li *et al*. [44] reported that the color temperature of the LED lamp was around 3700K, and the color rendering index was 70. The color rendering of white LEDs using CaAlSiN_3_:Ce^3+^ is higher than that of LEDs using Ca- or Li-α-sialon:Eu^2+^, which is ascribed to the broader emission band of CaAlSiN_3_:Ce^3+^.

### 4.2. Multi-phosphor-converted LEDs

1-pc white LEDs usually have low color rendering indices (Ra < 80), which are not acceptable for general lighting. To improve the color rendering properties, two or three or even more phosphors should be applied to obtain broad emission spectra of white LEDs.

Xie *et al*. [47] used Ca-α-sialon:Yb^2+^ and Sr_2_Si_5_N_8_:Eu^2+^ phosphors as well as a blue LED (λ_em_ = 450 nm) to fabricate high color rendering white LEDs. The color rendering index of these LEDs was of Ra = 82. Mueller-Mach *et al.* [36] reported highly efficient white LEDs with Ra ~90, using the phosphor blend of Sr_2_Si_5_N_8_:Eu^2+^ and SrSi_2_O_2_N_2_:Eu^2+^. Yang *et al*. [48] reported white LEDs using SrSi_2_O_2_N_2_:Eu^2+^ and CaSiN_2_:Ce^3+^, which showed the color rendering index of Ra = 92. Fukuda *et al.* [38] attempted to fabricate white LEDs using Sr_3_Si_13_Al_3_O_2_N_21_:Eu^2+^ and (Sr,Ca)_2_SiO_4_:Eu^2+^, the color rendering index of which was in the range of 82–87. 

Three-phosphor-converted white LEDs, using β-sialon:Eu^2+^, Ca-α-sialon:Eu^2+^ and CaAlSiN_3_:Eu^2+^ and a blue LED chip, were reported by Sakuma *et al*. [49]. The color rendering index (Ra) varies in the range of 81–88.

**Figure 7:**
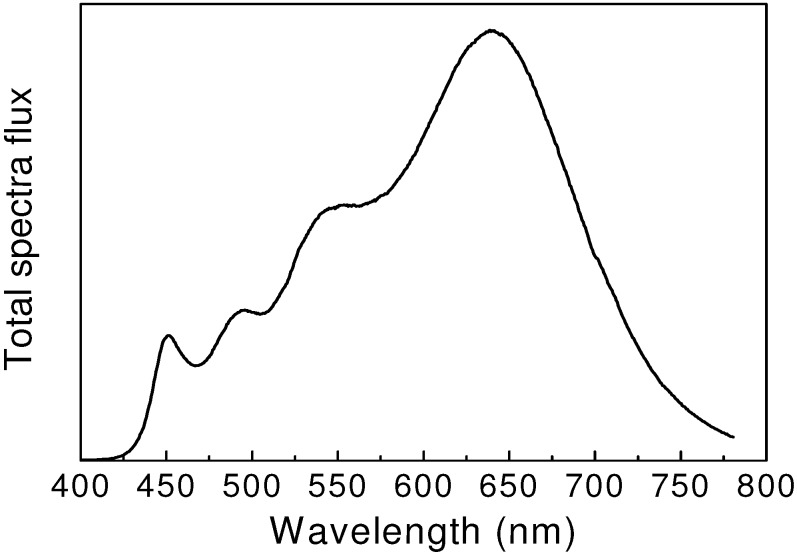
Emission spectrum of 4-pc white LEDs. The color rendering index Ra is 96, and the color temperature is 2800 K.

Ultrahigh color rendering white LEDs were obtained by using four phosphors. Kimura *et al.* [50] reported that, the color rendering index could reach 98 by combining BaSi_2_O_2_N_2_:Eu^2+^, β-sialon:Eu^2+^, Ca-α-sialon:Eu^2+^ and CaAlSiN_3_:Eu^2+^ with a blue LED (see Figure 7). Alternatively, Takahashi *et al.* [26] fabricated 4-phosphor-convereted white LEDs by applying JEM:Ce^3+^, β-sialon:Eu^2+^, Ca-α-sialon:Eu^2+^ and CaAlSiN_3_:Eu^2+^ with a near UV LED chip (λ_em_ *=* 405 nm). These white LEDs show the color rendering index of Ra *=* 96.

Besides their applications in general lighting, white LEDs are also promising alternatives to cold cathode fluorescent lamps (CCFL) for backlights in liquid crystal displays (LCDs). Xie *at al*. [51] reported a wide color gamut white LED backlight for LCDs. It was produced by using β-sialon:Eu^2+^, CaAlSiN_3_:Eu^2+^ and a blue LED. The color gamut of this three-band white LED can reach 92% of the NTSC standard, which is superior to that of CCFL (usually 65–75%).

## 5. Summary and Outlook

Rare earth doped nitride phosphors have been emerged as a new class of luminescent materials, showing promising photoluminescent properties such as significantly red-shifted excitation and emission spectra, abundant emission colors, small thermal quenching, and high quantum efficiency. These interesting properties enable nitride phosphors to be very suitable for use as promising down-conversion luminescent materials in white LEDs with high color rendering index and/or tunable color temperatures. 

Nitride compounds, typically nitridosilcates, nitridoaluminosilicates or oxonitrido(alumino) silicates, have been demonstrated to be good host lattices for luminescent materials. The versatile crystal structures of oxynitride/nitride materials offer great opportunities to produce a variety of emission colors when doped with Eu^2+^ or Ce^3+^ ions with the 5d → 4f electronic transition. Although strong luminescence is observed in some nitride phosphors such as β-sialon:Eu^2+^ and AlN:Eu^2+^, either the location of Eu^2+^ in the lattice or the local structure surrounding Eu^2+^ is not fully understood. This requires a deep investigation of the fine structure of doped materials by taking advantages of analytic techniques like EXAFS, HRTEM, XPS, and simulations. In comparison with oxide phosphors, the synthesis of nitride ones is usually costly and ineffective. Moreover, it is very hard for traditional synthetic methods to reduce the nitride particle size down to several nanometers. It is thus necessary to develop novel methods to produce nitride phosphors cost-effectively or to obtain nano powders with high luminescence.

As a new class of luminescent materials, nitride phosphors are attracting significant attentions due to their great suitability in white LEDs. Thus, searching for new nitride crystals, especially covalent multinary nitride crystals, will be continuously pursued. In addition, it will be an interesting topic for developing nitride phosphors as wavelength-conversion layers in silicon solar cells to enhance the light-harvesting efficiency.
